# New Appliances and Things Medical

**Published:** 1902-07-26

**Authors:** 


### NEW APPLIANCES AND THINGS MEDICAL.

[We shall be glad to receive at our Office, 28 & 29 Southampton Street, Strand, London, W.C., from the manufacturers, specimens of all new preparations and appliances which may be brought out from time to time.]

### BILLONS OVO-LECITHINE.

(London Depôt, 16 Water Lane, Great Tower Street, London, E.C.)

The value of Lecithine in the treatment of nervous disease, and for the restoration of exhausted nerve cells, is now generally acknowledged. As an organic and natural combination of phosphorus it constitutes the most rational medium for the exhibition of this element. To ensure the direct and immediate assimilation of phosphorus by the system ovo-lecithine is most happily adapted. It is prepared from the egg, in which combined lecithine exists in considerable percentage. Ovo-lecithine can be administered in three forms, which forms have been the subject of special study with a view to preventing the possibility of any alteration in the chemical constitution. These three forms are pills, which are most applicable for adults; a granulated form dissolved in water or milk and convenient for children; and a hypodermic injection in cases where a rapid effect is desired.

### MARVIS.

(Patent Fish Food Syndicate, Ltd., Eastcheap, London, E.C., and Wick, N.B.)

Marvis is a dried preparation made from the muscle tissue of fish. An examination of the chemical analysis of Marvis suggests that this new food preparation has very valuable properties as a nutritive substance—it contains over 50 per cent. of proteids and a high percentage of phosphorus (0·31 per cent.). The nutritive value of the flesh of fish is appreciated by dietetists for the reason that, as compared with that of animal flesh, it contains but a small percentage of extractive or purine bodies, which are now generally recognised as being contra-indicated for persons of gouty habit or weakly constitution, and further, it is probable that the organic combinations of phosphorus contained in the tissue of fish are of value in building up impaired or weakly nerve structures. For invalids generally, and especially for those suffering from neurasthenic or nervous symptoms, Marvis is strongly recommended. Marvis is a white, or very slightly yellow, powder, with the taste and flavour peculiar to fish. When mixed into a thin paste with milk, water, or other fluid, it constitutes a highly-nutritive food for invalids or children.

### ODOL—A NEW ANTISEPTIC DENTIFRICE.

This new antiseptic, called Odol, is a chemical body prepared from coal tar and closely related to salol, from which, however, it differs in its physical constitution and unstable combination. Odol, or odolol, is a golden-yellow, viscous oil, insoluble in most liquid media, but forming an opaque emulsion when mixed with water. When in contact with organic material it readily breaks up into salicylic acid and phenol. And these bodies, liberated in a free and nascent condition, exercise in a peculiarly effective manner the antiseptic action which belongs to each individually. The value of a chemical body of these properties is self-evident in cases in which the continued action of an antiseptic is required. Odol, in the presence of organic matter, will continue to decompose into its component antiseptic elements as long as there is material on which to exercise its chemical action. Hence the value of Odol as a mouth-wash; its cleansing and deodorising action is continued as long as there is any Odol left to operate or as long as there is any organic matter which requires treatment. Odol is supplied in very ingenious flasks which are well adapted for toilet use. We commend the use of Odol to those who appreciate the advantages of oral asepsis.

